# Ground Reaction Force and Centre of Pressure During the Golf Swing and Associations with Clubhead Speed and Skill Level: A Systematic Review

**DOI:** 10.1007/s40279-025-02391-3

**Published:** 2026-02-07

**Authors:** Andrew Watson, Andrew Murray, Alex Ehlert, Jiaqing Xu, Steve Williams, Abbie Spiegelhalter, Daniel Coughlan, Anthony Turner, Chris Bishop

**Affiliations:** 1 London Sport Institute, Faculty of Science and Technology, Middlesex University, London, England; 2 The R&A, St. Andrews, Scotland; 3 European Tour Group, Virginia Water, Surrey, England; 4 Ladies European Tour, Buckinghamshire Golf Club, Buckinghamshire, England; 5 Independent Researcher, Knightdale, NC USA; 6 Sports Academy PGA Frisco, PGA of America, Frisco, TX USA; 7 England Golf, Woodhall Spa, Lincolnshire, England

## Abstract

**Background:**

Optimising performance with a driver, fairway woods and long irons is a key focus for many golfers, with the primary goal of hitting the ball as far as possible while maintaining the ball in play. As such, recent years have seen a strong focus in the golf literature on how to maximise performance off the tee by investigating the factors that lead to the increase in clubhead speed (CHS). These factors include: (i) effective use of ground reaction force (GRF) during the swing and (ii) the path followed by the centre of pressure (CoP).

**Objectives:**

The primary purpose of this systematic review was to investigate GRF and CoP within golf research and to identify what associations they have with CHS and skill level.

**Methods:**

This systematic review followed the most recent Preferred Reporting Items for Systematic Reviews and Meta-Analyses (PRISMA) 2020 guidelines. In total, 129 studies where initially retrieved from SPORTDiscus, Medline and CINAHL databases, with studies meeting the inclusion criteria being subject to Newcastle–Ottawa’s quality assessment criteria (with a maximum score of 9 being possible).

**Results:**

A total of 24 studies met the inclusion criteria for this review. Nine empirical investigations showed moderate-to-strong relationships between either: (i) CoP and CHS or (ii) GRF and CHS. In addition, more skilled golfers tended to exhibit higher GRF and superior CHS than less skilled golfers. From a quality assessment standpoint, all 24 studies scored either a 7 or 8.

**Conclusions:**

Changes in both CoP and GRF represent important factors which contribute to superior golf performance, as defined by increases in CHS or reduced handicaps. Clearly defined methods for assessing force during the golf swing and universal terminology regarding GRF and CoP metrics are recommended for further research.

## Key Points

**Table:** 

Optimising performance with a driver, fairway woods and long irons is a key focus for many golfers, with the primary goal of hitting the ball as far as possible while maintaining the ball in play.
As such, recent years have seen a strong focus in the golf literature on how to maximise performance off the tee by investigating the factors that lead to the increase in clubhead speed (CHS). These factors include: (i) effective use of ground reaction force (GRF) during the swing and (ii) the path followed by the centre of pressure (CoP).
Nine empirical investigations showed moderate-to-strong relationships between either: (i) CoP and CHS or (ii) GRF and CHS. In addition, more skilled golfers tended to exhibit higher GRF and superior CHS than less skilled golfers.
Changes in both CoP and GRF represent important factors which contribute to superior golf performance, as defined by increases in CHS or reduced handicaps. Clearly defined methods for assessing force during the golf swing and universal terminology regarding GRF and CoP metrics are recommended for further research.

## Introduction

Golf is a popular sport with > 100 million people participating globally from over 80 countries [1]. Optimising performance with the driver is a key focus for many players [2], with the primary goal of hitting the ball as far as possible while maintaining the ball in play [3]. While the driver swing (the club used to generate maximum speed and distance off the tee) is a highly coordinated and individual motion [4], generating the fastest clubhead speed (CHS) via effective force transfer through the kinematic chain is one of the main goals [5]. As such, there has been a focus in the golf literature on how to maximise performance off the tee (i.e. distance and accuracy) by investigating the factors that lead to the increase in CHS. These factors include: (i) effective use of ground reaction force (GRF)—the force acting on the body from the ground during the swing [2], and (ii) the path followed by the centre of pressure (CoP), often referred to as weight transfer within golf coaching [3, 6]. Consequently, the more widespread use of technologies such as force or balance platforms has allowed both GRF and CoP data to be better quantified and analysed during the golf swing [7].

Analysis of GRF has been described as a vital method for investigating the relationship between the mechanical motion of an object and the forces acting on the object [8]. Force is expressed in three directions: (i) vertical (*Z*), (ii) medio-lateral or horizontal (*Y*), and (iii) anterior–posterior (*X*). Specifically, golfers who can generate higher vertical GRF will often benefit from the proximal-to-distal sequencing that occurs during the swing, enabling greater energy transfer through the kinetic chain to the club. However, it should be acknowledged that effective use of GRF in the swing is also dependent on timing, not just solely on the magnitude of force produced. The relevance here is that as players become better and more efficient at this, they will generate faster CHS and improve their shot distance [3, 9]. In turn, when a golfer is able to improve their distance (especially off the tee or on holes where a wood is deemed safe to use—i.e. on par 4 or 5 holes), they may be able to ‘gain strokes’ and a competitive advantage on their opponents during tournaments [10]. Therefore, it is not surprising that CHS has been shown to be strongly associated with handicap (*r* = 0.95), and thus, a good proxy for golf performance [11].

Numerous studies have investigated the role of GRF in golf performance. For example, Dale and Brumitt [12] have previously shown that both full and partial swings can produce compressive forces in the spine (calculated via inverse dynamic algorithms derived from force platform and kinematic data) over seven times a player’s bodyweight. When focused more on golf performance, Barrentine et al. [13] suggested that the interaction between the ground and the lower body plays a pivotal role in increasing CHS and overall swing power. Similarly, Williams [14] found that peak horizontal GRF was associated with CHS across golfers of varying handicaps (*r* = 0.69). Additionally, Bourgain et al. [15] highlighted the importance of horizontal GRF and its relationship to CHS, particularly when considering the sum force from both feet (*r* = 0.83). Collectively, these data show that GRF plays a critical role in contributing to a golfer’s CHS, which, in turn, may contribute to enhanced performance statistics on the course [10].

CoP is defined as the location where the resultant GRF vectors (combined *x* and *y*) would act if it were to have a single point of application [16]. Several studies have also explored the relationship between weight transfer and performance in golf. Koslow [17] reported that 84% of novice golfers failed to demonstrate the “normal” weight transfer pattern recommended by golf coaches (a shift in weight onto the lead leg before impact), often resulting in a reverse pivot or incomplete weight shift. In contrast, experienced golfers typically exhibit a more complete and effective weight transfer during their swings [18, 19]. However, the small sample sizes in these abovementioned studies limit the ability to generalise their findings to the wider golfing population. Richards et al. [20] found no significant differences in weight transfer between high and low handicap golfers. Conversely, Robinson [21] observed a moderate association between weight transfer and CHS at ball contact across golfers with varying skill levels (*r* = 0.48). Finally, Mason et al. [22] failed to find similar correlations among single-figure handicapped golfers (*r* = 0.28), suggesting that weight transfer’s impact on performance may depend on factors such as skill level or swing style.

**Fig. 1:**
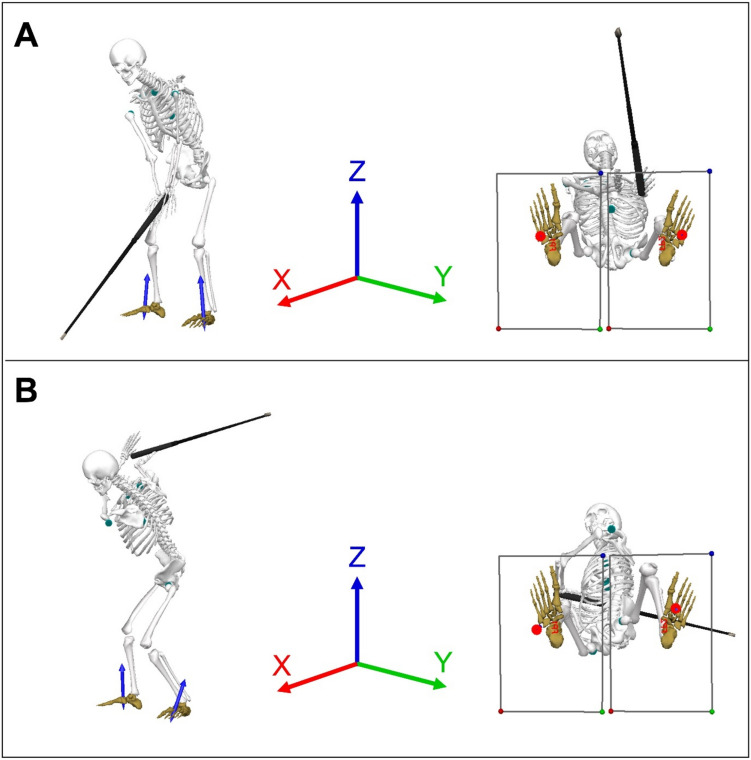
Illustration of ground reaction force (GRF) vectors, centre of pressure (CoP) positions and body weight shift across key phases of the golf swing. Visual three-dimensional (3D) models show the address position (**A**) and the top of the backswing (**B**). The global coordinate system (*X* = red, *Y* = green, *Z* = blue) defines the mediolateral, anteroposterior and vertical directions used to describe GRF components. Additional blue arrows represent the GRF vectors originating from each foot, indicating both the magnitude and direction of the external forces acting on the golfer. Red dots mark the CoP, showing the point of application of the resultant GRF vector within each foot. Across the swing phases, the CoP under the back foot shifts laterally, while the front foot CoP moves anteriorly towards the forefoot

Despite numerous studies linking weight transfer and GRF to CHS, there is still uncertainty regarding the strength and consistency of these relationships. Variability in findings may stem from differences in player skill levels, natural fluctuations in swing variability, coaching received, experimental methods, and sample sizes. However, it is generally agreed that achieving effective weight transfer and maximizing foot–ground interactions play crucial roles in generating higher CHS and improving performance in golf. Therefore, the purpose of this systematic review is to investigate GRF and CoP within golf research and to identify what association they have with CHS and skill level (as defined by handicap). Importantly though, golf biomechanics literature to date has utilised a wide variety of methods (and even terminology); thus, to help the reader contextualise some of the specific terms used herein, Table 1 provides operational definitions for relevant terms to ensure that discrete differences between study designs are understood. Further to this, Fig. 1 provides an illustration of these concepts when undertaking a biomechanical analysis of the golf swing.

**Table 1:** Operational definitions for key terms in this systematic review

Terminology	Operational definition
Ground reaction force (GRF)	Described as vertical, mediolateral or anteroposterior, which are the three components of the GRF. When applicable, GRF measures will be described as being directed towards or away from the target (*y*-axis) for mediolateral GRF, and towards the toes or heel (*x*-axis) for anteroposterior GRF. Studies differ in terms of the exact GRF measurements calculated and may include the magnitude of GRF at a specific time point or swing position, or the peak magnitude of GRF in terms of absolute or relative force. When possible, the specific measure of GRF will be specified within the results and discussion
Centre of pressure (CoP)	The location where the resultant GRF vectors (combined *x* and *y*) would act if it were to have a single point of application [16]. The position of the CoP can be described within mediolateral (position relative to the trail and lead foot on the *y*-axis) and anteroposterior (position relative to the toe and heel of the foot on the *x*-axis) vectors. Measures of CoP motion during the swing include the range (e.g. distance covered by the CoP) and velocity
Weight shift	Defined as the transfer of body weight (force) during the swing, often quantified as displacement of the centre of mass (CoM) of the body or body–club system. Weight shift and CoP motions are often related, but not interchangeable. For this reason, studies which describe CoP or weight shift may be discussed together, but the specific measure will be defined when possible

## Methods

This review followed the Preferred Reporting Items for Systematic Reviews and Meta-Analyses (PRISMA 2020) guidelines [23] but was not registered with the International Prospective Register of Systematic Reviews (PROSPERO). These guidelines are an update from the 2009 PRISMA guidelines and reflect advancements in the methods to identify, select, appraise and synthesise included studies. This includes a 27-item checklist, and the structure and presentation of these items have been modified to facilitate easier implementation by researchers.

### Inclusion and Exclusion Criteria

For inclusion in this systematic review, studies were required to: (i) be original empirical investigations, (ii) involve populations aged between 16 and 65 years old and (iii) include male or female subjects. Studies were excluded if they were: (i) not published in English, (ii) not peer reviewed, (iii) previously published systematic or scoping reviews or (iv) included subjects with physical disabilities (given that this was not the purpose of this review).

### Search Strategy

Searches were performed in SPORTDiscus, Medline and CINAHL databases using EBSCOhost by the lead investigator (A.W.), with all searches taking place in September 2024. To keep the search criteria as inclusive as possible, the descriptors used were: ‘ground reaction force’ and ‘centre of pressure’, with searches filtered using the abovementioned exclusion criteria. The final structure of words with the descriptors and operators together was (ground reaction force) OR (centre of pressure) AND (golf). Each database used has filters to allow for both inclusion and exclusion criteria to be applied to the search to reduce unmatched and unrelated articles in the results. Within the EBSCOhost dashboard, special limiters were selected for each database. For SPORTDiscus, the special limiters were Language—English, Country—All and Academic Journal for Publication Type. For CINAHL, the limiters selected were Language—English, Species—Human, Journal Subset—Peer Review, Publication Type—Journal Article, Sex—All and Age Groups—Adolescent (13–18 years), Adult (19–44 years), Middle Aged (45–64 years). For Medline, the special limiters were set to Language—English, Species—Human, Sex—All, Age Related—Adolescent (13–18 years), Adult (19–44 years), Middle Aged (45–64 years) and Publication Type—Journal Article.

### Data Extraction

The results were then screened by the lead investigator (A.W.) and a second author (C.B.) by evaluating titles and abstracts of each article and excluding those that: (i) did not fit the inclusion or exclusion criteria, (ii) did not measure GRF or CoP or (iii) the full text was unavailable. A third author (A.E.) was then consulted if there were any disputes on the inclusion or exclusion of studies. The remaining articles were read in full and charted using Microsoft Excel. The studies where charted under the following headings: Author, Title, Purpose, Design, Methods, Results, Strengths, Weaknesses and Conclusion.

### Quality Assessment

The quality of included studies was evaluated by two authors (A.W. and C.B.) using the Newcastle–Ottawa Scale (NOS) for cohort studies [24]. If any disagreements were evident, a third reviewer (A.E.) was included to finalise a decision. As all included studies were non-randomised studies, this quality assessment was chosen post hoc as the most appropriate quality assessment tool [25]. All studies were scored in three areas: (i) selection, (ii) comparability and (iii) outcome. A maximum score of four, two and three for each respective area was possible, resulting in a maximum possible score of nine.

## Results

### Descriptive Analysis

**Fig. 2:**
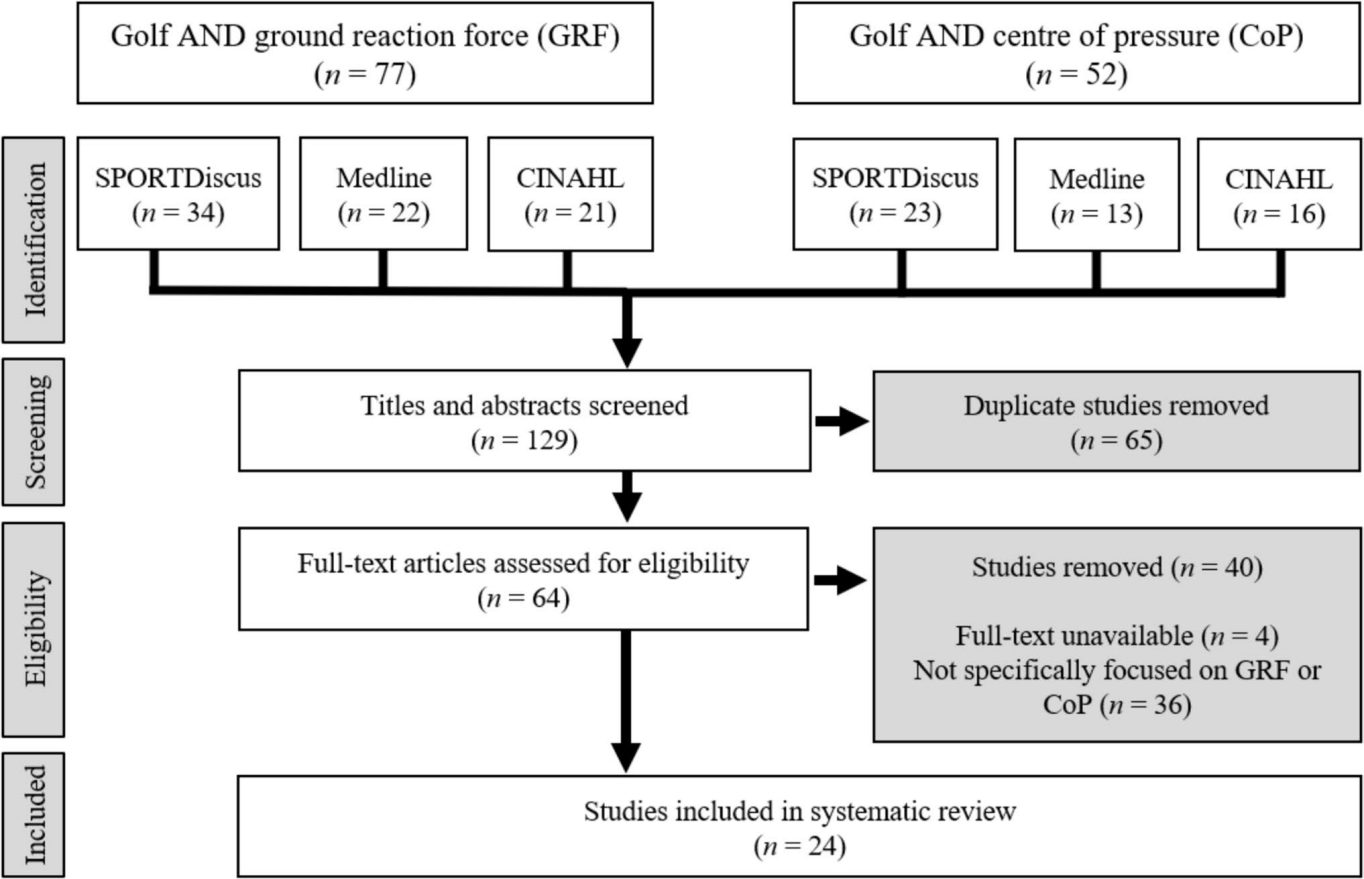
Flow diagram of the charting process, based on the PRISMA 2020 guidelines [23]

In total, 129 studies where initially retrieved from the three databases. After screening the titles, abstracts and full texts, 24 studies met the inclusion criteria for this review. Figure 2 shows the phases of the charting process based on PRISMA 2020 guidelines [23]. A summary of the correlational design studies included is presented in Table 2 with all non-correlational design studies presented in Table 3. Table 4 outlines the group scoring for all studies using the Newcastle–Ottawa System (NOS) for each study. All studies were of an acceptable standard, with none measuring below a minimum of seven on the NOS scale; however, no studies reached the maximum score of nine.

**Table 2:** Overview of methods in studies reporting associations between ground reaction force (GRF), centre of pressure (CoP) and clubhead speed (CHS) or handicap

Study	Title	Objective	Methods	Results
Ball and Best [26]	Different centre of pressure patterns within the golf stroke I: cluster analysis	To determine whether different weight transfer styles exist in the golf swing by applying cluster analysis to centre of pressure (CoP) patterns	Sixty-two male golfers aged 34 ± 14 years (mean ± SD), ranging from professionals to high handicap golfers (handicap 11 ± 8) and recreational golfers, performed ten simulated golf drives using their own driver, hitting a golf ball into a net placed 3 m away while standing on AMTI force plates	Association between CHS and CoP range (*r* = 0.53, *p* = 0.001)
Ball and Best [27]	Different centre of pressure patterns within the golf stroke II: group-based analysis	Evaluated the relationship between centre of pressure measures and club head velocity within the ‘front foot’ and ‘reverse’ styles	Thirty-nine front foot golfers and 19 reverse golfers performed swings with a driver while standing on two force plates. From the force plate data, centre of pressure displacement, velocity, range and timing parameters were calculated	CoPy range in metres and maximum CoPy velocity were strongly correlated with CHS (*r* = 0.54, *p* < 0.001) Stance width (distance between the feet) was correlated with CHS (*r* = 0.47, *p* < 0.005)
Ball and Best [29]	Golf styles and centre of pressure patterns when using different golf clubs	To establish whether two swing styles are also evident when using other clubs, and whether golfers use the same swing style when using different clubs	Forty-six professional and amateur golfers performed swings to hit a ball into a net placed 3 m away. Ten swings were performed for each of the driver, 3-iron and 7-iron while standing on two force plates. The position of the golfer’s centre of pressure parallel with the line of shot and relative to the feet was quantified at eight swing events that were identified from 200-Hz video	From this analysis, both the point biserial correlation values (driver: *r*^pbi^ = 0.61; 3-iron:* r*^pbi^ = 0.54; 7-iron:* r*^pbi^ = 0.63) and *C*-index (driver: 1.08; 3-iron: 1.09; 7-iron: 1.02) indicated the two-cluster solution was unambiguously optimal for all clubs
Ball and Best [30]	Centre of pressure patterns in the golf swing: individual-based analysis	The aim of this study was to extend this work by examining the importance of weight transfer in the golf swing on an individual basis	Five golfers (professional and amateur) performed 50 swings with the driver, hitting a ball into a net. The golfer’s centre of pressure position and velocity, parallel with the line of shot, were measured by two force plates at eight swing events that were identified from high-speed video. The relationships between these parameters and club head velocity at ball contact were examined using regression statistics	Larger values of mediolateral CoP range were associated with larger CHS (*R*^2^ = 0.20–0.52, *p* < 0.001) for ‘average within-golfer relationships’. *Note:* data pooled for only three golfers in subsequent analysis
Bourgain et al. [31]	Contribution of vertical and horizontal components of ground reaction forces on global motor moment during a golf swing: a preliminary study	To evaluate the contribution of vertical and horizontal components of ground reaction forces to global motor moment during the swing	Three recreational (golf handicaps of 19.5, 12 and 7) and three professional golfers (professionals for 2 months, 5 years and 9 years) participated in this study. Subjects performed 20 golf swings with their own driver club, in an indoor motion analysis laboratory. Each swing was tracked with a dedicated launch monitor (TrackMan 3, Trackman golf), measuring the ball flight and simulating its trajectory	Association between CHS and vertical + horizontal force combined (*R*^*2*^ = 0.70)
Bourgain et al. [15]	Effect of horizontal ground reaction force (HGRF) during the golf swing: implications for the development of technical solutions of golf swing analysis	To investigate the role of HGRF during the golf swing at the motor moment	Twenty-eight golf players were recruited and performed ten swings with their own driver club, in a motion analysis laboratory, equipped with a full body marker set. GRF was measured with force plates. A multibody kinematic optimization was performed with a full body model to estimate the instantaneous location of the golfer’s centre of mass (CoM). Moments created by the GRF at the CoM were investigated	Motor moment (calculated as the global moment produced by the GRF of both feet at the CoM) produced a strong correlation to CHS (*r* = 0.83)
Chu et al. [2]	The relationship between biomechanical variables and driving performance during the golf swing	Identify the variable important to driving ball velocity	A total of 308 golfers (266 males, 42 females) (mean ± SD: age 43.2 ± 15.6 years; height 1.77 ± 0.17 m; mass 83.5 ± 17.0 kg; USGA handicap 8.4 ± 8.4). 3D motion capture plus Kistler force plates	The ball velocity measured (61.0 ± 8.7 m s^−1^ or 136.5 ± 19.4 mph) correlated negatively with handicaps (*r* = − 0.71)
Han et al. [28]	Effects of the golfer–ground interaction on CHS in skilled male golfers	Golfer–ground interactions during the swing and associations between force/moment parameters and maximum CHS	Sixty-three highly skilled male golfers (handicap ≤ 3) performed shots in three club conditions (driver, 5-iron and pitching wedge), which were captured by an optical motion capture system and two force plates. In addition to the GRF, three different golfer–ground interaction moments (GRF moments, pivoting moments and foot contact moments) were computed	Combined backward (sagittal) forces (*r* = 0.32 for driver; *r* = 0.21 for 5-iron; *r* = 0.31 for pitching wedge) Lead-foot backward (sagittal) force (*r* = 0.46 for driver; *r* = 0.44 for 5-iron; *r* = 0.55 for pitching wedge) Trail-foot forward (sagittal) force (*r* = 0.29 for driver; *r* = 0.31 for 5-iron; *r* = 0.42 for pitching wedge) Lead-foot away (transverse) force (*r* = 0.45 for driver; *r* = 0.25 for 5-iron and pitching wedge) Combined maximum forces (*r* = 0.33 for driver; *r* = 0.33 for 5-iron; *r* = 0.35 for pitching wedge)
Jones et al. [6]	Centre of pressure golf swing movement strategies are better defined using a continuous approach than by segregated styles	To examine CoP data by a continuum and to determine relationships between CoP, handicap and CHS using a continuous approach	Centre of pressure paths of driver and 5-iron shots from 104 amateur golfers were analysed using discrete and continuous methods. Discrete methods used different cluster evaluation criteria, which resulted in 2-cluster and 20-cluster solutions being considered ‘optimum’	CoP (mediolateral) to CHS: *R*^2^ = 0.54 (driver), 0.56 (5-iron) CoP (mediolateral) to handicap: *R*^2^ = 0.34 (driver), 0.35 (5-iron)
McHugh et al. [32]	Kinematic, kinetic, and temporal metrics associated with golf proficiency	The purpose of this study was to determine which metrics identified golf proficiency	Kinematic, kinetic and temporal metrics and their sequencing were collected for shots performed with a driver by 33 male golfers categorized as proficient, average or unskilled (based on a combination of handicap, ball velocity and driving distance). Kinematic data were collected with high-speed motion analysis, and GRF data were collected from dual force plates	Proficient golfers GRF trail leg (% body mass) 78.3 ± 6.0; lead leg 139.5 ± 19.6. Average golfers trail leg = 81.8 ± 6.6; lead leg = 136.1 ± 24.2 Peak lead foot GRF to ball speed (*R*^2^ = 0.85) Peak lead foot GRF to driving distance (*R*^2^ = 0.74)

Front foot: style of golf swing where the CoP is mostly on the lead foot; reverse foot: style of golf swing where the CoP is mostly on the trail foot; point biserial correlation: a statistical measure to determine the strength and direction of the linear relationship between a binary variable and a continuous variable

*CHS* club head speed, *CoP* centre of pressure, *SD* standard deviation, *r* coefficient of variance, *R*^*2*^ coefficient of determination, *CoPy* centre of pressure on the *y*-axis

**Table 3:** Overview of methods reporting cross-sectional data for ground reaction force (GRF) and centre of pressure (CoP) in golf studies

Study	Title	Objectives	Design	Methods	Results
Blenkinsop et al. [33]	The effect of uphill and downhill slopes on weight transfer, alignment and shot outcome in golf	To examine changes in weight transfer, alignment and shot outcome during golf shots from flat, uphill and downhill slopes	Experimental	Twelve elite male golfers hit 30 shots with a 6-iron from a computer-assisted rehabilitation environment (CAREN) used to create 5° slopes while collecting 3D kinematics and kinetics of the swing. A launch monitor measured performance outcomes of both club and ball	Mean CoP 9.4% closer to the front foot for the downhill slope Mean CoP 8.9% closer to the back foot for the uphill slope CoP pattern remained unchanged between the three different conditions Repeated measures one-way analysis of variance (ANOVA) showed that there was a significant difference (range ***ƞ***^2^ = 0.69–0.96, *p* < 0.001) between the position of the CoP at each swing event
Choi et al. [34]	Improved determination of dynamic balance using the centre of mass and centre of pressure inclination variables in a complete golf swing cycle	Evaluated the ability for dynamic balance during a golf swing by using the centre of mass (CoM)–centre of pressure (CoP) inclination variables	Cohort trial	Twelve professional and 13 (1–14 handicap) and 10 (≥ 15 handicap) golfers participated in this study. Six infrared cameras and two force platforms were used to measure the net CoM and CoP trajectories. To evaluate dynamic balance ability, the CoM–CoP inclination angle, CoM–CoP inclination angular velocity and normalised CoM–CoP inclination angular jerk were used	Professional players’ inclination angle (6.13 ± 2.4º) from lead to trail foot during downswing was significantly different to both amateur groups (*p* < 0.01)
Jones et al. [35]	Differences in the structure of variability in ground reaction force trajectories provide additional information about variability in the golf swing	A novel measure of variability to ground reaction force (GRF) trajectories and highlights the use of such measures	Experimental	The variability and regularity of GRF trajectories were quantified for iron and driver shots from three participants with different skill levels. Pointwise median absolute deviation (p-MAD) was used to indicate the variability of GRF trajectories across their length and two alternative methodologies: sample entropy (SampEn) and cross-sample entropy (Cross SampEn) were used to determine their regularity	Wilcoxon rank-sum tests revealed a statistically significant difference in cross-SampEn between the left and right feet (Ws = 707,558, ***z*** = 5.42, ***α*** < 0.001, *d* = 0.13) Cohen’s effect size descriptors suggested no effect between the two feet No significant difference between SampEn scores between the left and right feet (Ws = 361, ***z*** = 0.89, ***α*** = 0.389, *d* = 0.15). Regarding the driver and the iron clubs, there was no significant difference in cross-SampEn values (Ws = 673,022, ***z*** = 1.76, ***α*** = 0.079, *d* = 0.04) or SampEn scores (Ws = 347, ***z*** = 0.44, ***α*** = 0.673, *d* = 0.07) between the two clubs
Jones et al. [36]	The relationship between skill and ground reaction force variability in amateur golfers	The ground reaction force variability in 104 amateur golfers for shots with drivers and 5-irons	Experimental	Principal component analysis was used as a data-reduction technique and allowed all three components of ground reaction force to be considered together	There were statistically significant trends for the higher skilled golfers to display lower variability in two of the five principal components (driver) and four of the five principal components (5-iron)
Kim et al. [37]	Biomechanical effects of ball position on address position variables of elite golfers	To investigate address position variables in response to changes in ball position in golfers	Experimental	Eleven male professional golfers were instructed to perform their golf swing. A 3D motion analysis system, with eight infrared cameras and two force platforms, was used to capture the address positions	When the ball was moved to the left, the left foot vertical GRF increased by 54.4% BW (± 4.3) versus 53.4% BW (± 5.3) When the ball was moved to the right, the CoP moved toward the right foot by − 8.4 ± 7.1 mm versus 11.5 ± 12.2 mm
Kim et al. [5]	Small changes in ball position at address cause a chain effect in golf swing	How ball position along the mediolateral (M-L) direction impacts ground reaction force, body segment and joint angles, and whole-body centre of mass during the golf swing	Experimental	Twenty professional golfers were asked to complete five straight shots for each five different ball positions along M-L: 4.27 cm (ball diameter), 2.14 cm (ball radius), 0 cm (reference position at preferred ball position), –2.14 cm and –4.27 cm, while their GRF and body segment motions were captured	Vertical GRF on left foot increased (left full (LF): + 1.8% BW and left half (LH): + 1.0% BW) while left-sided ball positions and right-sided positions were smaller (right half (RH): − 1.1% BW and right full (RF): − 1.6% BW) The opposite was observed for vertical GRF of the right foot (LF: − 2.3% BW, LH: − 1.3% BW, RH: + 1.1% BW, and RF: + 1.6% BW) in the left-sided and right-sided ball positions, respectively, compared with reference position (R0) (60.7 ± 4.7% BW, *p* < 0.01) Regarding mediolateral CoP, CoPy showed shifts towards and away from the target in left-sided and right-sided ball positions, respectively, between address and early backswing, compared with R0 (*p* < 0.01), and reappeared during downswing and impact (*p* < 0.01) In anteroposterior CoP, CoPx showed shifts towards posterior and anterior directions in left-sided and right-sided ball positions at impact compared with R0 (*p* < 0.01)
Kim et al. [38]	Golf swing in response to anteroposterior ball position	This study investigated the effects of ball position on golf-swing behaviours	Experimental	Twenty professional golfers performed golf swing at five different anteroposterior (forwards/backwards) ball positions: reference ball position (R0) and ± 2.14 cm (golf ball radius) and ± 4.17 cm (golf ball diameter) to the R0. Their swings were captured using a motion capture system with two force platforms. Statistical parametric mapping was used to compare the ground reaction force and swing kinematics for different ball positions	Significant differences in horizontal GRF were observed between the forward/backward (F/B) ball positions, particularly in the lead foot’s F/B GRF during early downswing (D225 to D135 – degrees of the shaft of the club throughout the downswing phase) (*p* < 0.001), trail foot’s F/B GRF during the back-to-downswing transition (TC) (*p* = 0.003), lead foot’s towards/away GRF during downswing (D225 to D135) (*p* < 0.001), and trail foot’s T/A GRF during late downswing and impact (D90 to I) (*p* = 0.001). These GRF values were lower in backward ball positions and higher in forward positions compared with R0. Significant differences were also found in the vertical GRF, with increased values in the forward ball positions: lead foot during early downswing (D225) (*p* = 0.003) and trail foot during late downswing and impact (D45 and I) (*p* = 0.002). Additionally, the trail foot’s F/B GRF during late downswing (D45) (*p* = 0.006) and the lead foot’s T/A GRF during impact (I) (*p* < 0.001) were significantly decreased for both backward and forward ball positions
Macadam et al. [39]	Wearable resistance acutely enhances club head speed in skilled female golfers	To explore the acute effects of wearable resistance on golf swing performance measures	Experimental	Five skilled female golfers (age = 22.0 ± 2.5 years, height = 163.1 ± 3.3 cm, body mass = 57.1 ± 3.4 kg and handicap 4 ± 1.2) who performed a series of golf shots with and without wearable resistance of 1.6 kg (2.8% body mass) attached laterally to the posterior trail side of the body. Flightscope launch monitor and force plate technology were used to quantify changes in club head speed and ground reaction force	Significant (*p* < 0.05) acute increases were found in club head speed (3.5%, *p* = 0.03), relative vertical ground reaction forces (11.4%, lead side, *p* = 0.01) and relative mediolateral ground reaction forces (7.1%, trail side, *p* = 0.03) with wearable resistance as compared with the unloaded condition
McNitt-Gray et al. [40]	Regulation of reaction forces during the golf swing	To determine how individual players regulate target and rear leg reaction forces when reducing shot distance using the same golf club (6-iron)	Experimental	Twelve skilled players hit golf balls using a 6-iron. Shot distance was varied by hitting the ball as they would normally and when reducing shot distance using the same club. During each swing, reaction forces were measured using dual force plates (1200 Hz) and 3D kinematics were simultaneously captured (110 Hz)	Peak resultant horizontal reaction forces of the target leg were significantly less than normal (5%, *p* < 0.05) when reducing shot distance. No significant differences in the orientation of the peak resultant horizontal reaction forces were observed
Okuda et al. [41]	Trunk rotation and weight transfer patterns between skilled and low skilled golfers	To examine trunk rotational patterns and weight transfer patterns that may differentiate swing skill level in golfers	Experimental	Thirteen skilled golfers (mean handicap 0.8 ± 2.6) and 17 low-skilled golfers (mean handicap 30.8 ± 5.5) participated in this study. Kinematic and kinetic data were obtained through high-speed 3D videography and force plates while the participant performed a full-shot golf swing with a driver	Lead foot VGRF (%BW) = backswing 35% (low skilled) versus 25% (skilled) Downswing 33% (low skilled) versus 59% (skilled) Trail foot VGRF (%BW) = backswing 76% (low skilled) versus 92% (skilled)
Peterson et al. [42]	Angular impulse and balance regulation during the golf swing	To determine how skilled players regulate linear and angular impulse while maintaining balance during the golf swing	Experimental	Eleven highly skilled players (< 5 handicap), 5 female and 6 male, all right-handed, hit 4–6 shots using a 6-iron and driver while on Kistler force plates	Relative net angular impulse (***N m s*** kg^**−**1^) generated by both the rear and target legs was greater for the driver (0.337) than for the 6-iron (0.291) Increases in net angular impulse with a driver involved increases in target leg resultant horizontal reaction force (RFh). Rear leg RFh angle was maintained between clubs, whereas target leg RFh became more aligned with the target line
Peterson and McNitt-Gray [43]	Regulation of linear and angular impulse during the golf swing with modified address positions	To determine how golfers coordinate their legs to regulate linear and angular impulse while modifying the lower-extremity address position during the swing	Experimental	Nine highly skilled golf players performed swings with a 6-iron under the normal, rear (trail) leg up and target (lead) leg up conditions. Components of linear and angular impulse generated by the rear and target legs (resultant horizontal reaction force, resultant horizontal reaction force angle and moment arm) were quantified and compared across the group and within a player	Net angular impulse did not change between conditions. Target leg angular impulse was greater in the target leg up condition than rear leg up condition. Regulation of linear and angular impulse generation occurred while increasing stance width and redirecting resultant horizontal reaction forces to be more parallel to the target line under modified address positions. Net linear impulse perpendicular to the target was near 0 or slightly posterior. Net linear impulse parallel to the target was less towards the target in the target leg up condition compared with normal and rear leg up conditions
Queen et al. [44]	Difference in peak weight transfer and timing based on golf handicap	To examine: (a) the changes in the peak ground reaction forces (GRF) and the timing of these events between high handicap (HHCP) and low handicap (LHCP) golfers and (b) the differences between the leading and trailing legs	Cross-sectional, control	Twenty-eight male golfers were recruited and divided on the basis of having a low handicap < 9 or high handicap > 9. Three-dimensional GRF peaks and the timing of the peaks were recorded bilaterally during a golf swing. The golf swing was divided into different phases: (a) address to the top of the backswing, (b) top of the backswing to ball contact and (c) ball contact to the end of follow through. Repeated measures analyses of variance (*α* = 0.05) were completed for each study variable: the magnitude and the timing of peak vertical GRF, peak lateral GRF and peak medial GRF (*α* = 0.05)	The LHCP group had a greater transfer of vertical force from the trailing foot to the leading foot in phase 2 than the HHCP group. The LHCP group also demonstrated earlier timing of peak vertical force throughout the golf swing than the HHCP group. The LHCP and HHCP groups demonstrated different magnitudes of peak lateral force. The LHCP group had an earlier timing of peak lateral GRF in phase 2 and earlier timing of peak medial GRF in phases 1 and 2 than the HHCP group. In general, LHCP golfers demonstrated greater and earlier force generation than HHCP golfers
Worsfold et al. [45]	A comparison of golf shoe designs highlights greater ground reaction forces with shorter irons	The current study aimed to compare forces generated at the shoe–turf interface when wearing different golf spikes	Experimental	Twenty-four golfers wore three different golf shoe traction designs (traditional metal spikes, alternative spikes and a flat-soled shoe with no additional traction) when performing shots with a driver, 3-iron and 7-iron. Ground reaction force was measured beneath the feet by two natural grass-covered force platforms	The maximum vertical force recorded at the back foot with the 3-iron and 7-iron was approximately 0.82 BW (body weight) and 1.1 BW at the front foot in both the metal spike and alternative spike golf shoe designs. When using the driver, these maximal vertical values were 0.49 BW at the back foot and 0.84 BW at the front foot. In the metal spike shoe, the vertical force generated at the back foot with both irons was 0.67 BW and at the front foot 0.96 BW with the 3-iron and 0.92 BW with the 7-iron. The back foot vertical force generated with the driver was 0.33 BW and at the front foot 0.83 BW while wearing the metal spike shoe. Results indicated greater force generation with the irons

*CoPx* centre of pressure on the *x*-axis, *p-MAD* pointwise median absolute deviation: a measure of variability calculated by finding the median of the absolute difference between each data point and the median of the dataset, *SampEn* sample entropy: measures the complexity or irregularity of a single time series, *cross SampEn* cross-sample entropy: measures the degree of asynchrony or dissimilarity between two different time series, *net angular impulse (N m s kg*^*−1*^*)* change in angular momentum caused by net torque acting over a period of time, *RFh* resultant horizontal reaction force, *BW* bodyweight, *CoP-CoM* centre of pressure to centre of mass, *F/B* forward/backward (anteroposterior direction), *T/A* towards/away (mediolateral direction)

**Table 4:** Newcastle–Ottawa quality assessment scoring system for included studies

Study	Selection (0/4)	Comparability (0/2)	Outcome (0/3)	Total (0/9)
*Associative study designs*				
Ball and Best [26]	**	**	***	7
Ball and Best [27]	**	**	***	7
Ball and Best [29]	***	*	***	7
Ball and Best [30]	***	*	***	7
Bourgain et al. [15]	***	*	***	7
Bourgain et al. [31]	***	*	***	7
Chu et al. [2]	***	*	***	7
Han et al. [28]	***	**	***	8
Jones et al. [6]	***	*	***	7
McHugh et al. [32]	****	*	***	8
*Group comparison or cross-over study designs*				
Blenkinsop et al. [33]	***	*	***	7
Choi et al. [34]	***	**	***	8
Jones et al. [35]	***	*	***	7
Jones et al. [36]	***	*	***	7
Kim et al. [37]	***	*	***	7
Kim et al. [5]	***	*	***	7
Kim et al. [38]	***	*	***	7
Macadam et al. [39]	***	*	***	7
McNitt-Gray et al. [32]	***	*	***	7
Okuda et al. [41]	****	*	***	8
Peterson et al. [42]	***	*	***	7
Peterson and McNitt-Gray [43]	***	*	***	7
Queen et al. [44]	***	*	***	7
Worsfold et al. [45]	***	*	***	7

### Overview of Methods

Ten studies (41.7%) were correlational by design (Table 2), reporting associations between GRF with either CHS or handicap, with the remaining 14 (58.3%) being a mixture of cross-sectional study designs (Table 3). The most investigated golf club was the driver, which was used in ten studies (41.7%). There were six (25%) studies that investigated multiple clubs, including driver and long and mid irons, and eight (33%) studies that only investigated a single iron (7, 6 or 5 iron).

Of the ten correlational studies, seven studies (29%) presented correlational data on CoP and its relationship to CHS, handicap, ball velocity or distance. Two studies (8%) investigated the relationship between CHS and GRF, and one study (4%) investigated the relationship between CHS and handicap. In total, four studies (16.7%) included less than 10 subjects, four (16.7%) included less than 20 subjects, ten (41.7%) included less than 50 subjects, three (12.5%) included between 50 and 100 subjects and three (12.5%) included over 100 subjects. While all studies used force plates, 13 (54%) also utilised three-dimensional (3D) motion capture; however, no standardised method for recording swing performance was used, with many studies relying on subjects completing ten shots with each club (where multiple clubs were used). Regarding the swing itself, there were varying methods used to identify timepoints or positions within the swing for analysis. Specifically, in both studies by Ball and Best [26, 27], the authors defined key phases in the swing to be: takeaway, mid backswing, late backswing, top of the backswing, early downswing, mid downswing, ball contact and mid follow-through. Han et al. [28] identified 11 swing points and referred to them as: break-away, mid backswing, late backswing—arm-based, late backswing, end of pelvis rotation, top of the backswing, early downswing—arm-based, early downswing, mid downswing, ball impact and mid follow-through. While a considerable overlap exists between these definitions, some differences likely lead to challenges when comparing data between methodologies. Two studies (8%) investigated the impact of hitting a ball from an uneven stance (i.e. with the lead foot higher than the trail foot) and its effects on GRF, one study (4%) investigated the impact of wearing a resistive vest during the swing and its effects on GRF and swing speed, and three studies (12.5%) investigated how ball position impacted kinematics and kinetics.

## Discussion

The aim of this systematic review was to identify associations within the literature between GRF and CoP to both CHS and skill level in golfers. In total, 24 studies were included in the final analysis, with 10 showing moderate-to-strong relationships between CoP and GRF to CHS. Further to this, more skilled golfers tended to exhibit higher GRF and superior CHS than less skilled golfers. When considering cross-sectional study designs (*n* = 14), a range of study designs and aims were examined. Of note, notable changes in CoP were evident when golfers played shots on downhill and uphill slopes; force production was greater when swinging a driver compared with irons; and skilled golfers exhibit a greater unweighting movement in their lead leg compared with less skilled players, enabling superior vertical GRF during the swing.

### Associative Study Designs

A study by Ball and Best [26] investigated the CoP in professional and recreational golfers and identified a moderate correlation between CoP range (i.e. the distance the CoP moved along the *y*-axis) and CHS (*r* = 0.53). This was further supported in a separate study by the same authors [27] who showed that CoP velocity along the *y*-axis was moderately correlated with CHS (*r* = 0.54). Further to this, stance width (the distance between the two feet during the address position) was significantly but moderately correlated with CHS (*r* = 0.47). These data support the notion that the distance between the two feet allows for a larger range of movement for the CoP along the *y*-axis, and moving this mass at a greater velocity results in an increased CHS. Further to this, the relevance of a golfer’s CoP appears to be the same between different clubs, as shown by Ball and Best [29], where the correlation values between CHS and different clubs remained relatively consistent for a group of 46 golfers (driver: *r* = 0.61; 3-iron:* r* = 0.54; 7-iron:* r* = 0.63). Even when swings were examined on a more individual basis, the mediolateral movement of CoP was still associated with CHS in a group of five golfers performing 50 swings with a driver (*R*^2^ = 0.30). Jones et al. [6] also reported strong associations between mediolateral CoP and CHS when using a driver (*R*^2^ = 0.54) and a 5-iron (*R*^2^ = 0.56), in 104 amateur golfers. They also reported correlations between CoP and handicap (driver: *R*^2^ = 0.34; 5-iron: *R*^2^ = 0.35). Collectively, and noting that some studies have reported correlations (*r* values), while others have reported the coefficient of determination (*R*^2^ values), the relevance of the CoP during the golf swing is clear, with a growing body of evidence.

When focusing on the relevance of GRF, Bourgain et al. [31] showed that combined vertical and horizontal forces explained a large amount of CHS variance (*R*^2^ = 0.70). In a separate study, Bourgain et al. [15] further developed this work by examining the importance of horizontal force production during the swing, which showed a very large correlation with ‘motor moment’ (*r* = 0.83), defined as the moment produced by the GRF of both feet at the CoM. Han et al. [28] performed an in-depth analysis of GRF and its interaction with CHS, reporting multiple correlations (Table 2) across three clubs for 63 male golfers – all with handicaps < 3. Of note, lead foot ‘backward force’ showed the strongest correlation with CHS (*r* = 0.55), but interestingly this was when using a pitching wedge. These data demonstrate how GRF can contribute to the development of CHS, while transferring energy through the kinetic chain, down the golf club to the ball, at impact. Finally, McHugh et al. [32] also reported a very large amount of ball speed and driving distance variance from the lead foot’s peak GRF (*R*^2^ = 0.85 and 0.74, respectively). Given the presented data from this group of studies, it seems prudent to suggest that both CoP and GRF have strong and potentially meaningful associations with developing faster CHS, which subsequently results in higher ball speeds and driving distance if the timing of these are used in an advantageous way during the swing.

### Group Comparison or Cross-Over Study Designs

The measurement of CoP has been utilised in a variety of ways during research to identify the strategy employed by golfers when performing different types of golf swings. For example, Choi et al. [34] investigated the changes in CoP and centre of mass (CoM) between 12 advanced, 13 amateur and 10 novice golfers to determine how dynamic stability strategies change between skill levels. The results showed a significant difference in inclination angle between the trail and lead foot during the downswing (6.13° ± 2.4°) between advanced and novice golfers. The authors suggested that this was an indication of improved dynamic balance in advanced golfers during the swing. Blenkinsop et al. [33] investigated changes in CoP in 12 elite golfers hitting 6-irons from simulated uphill and downhill lies. Interestingly, golfers manipulated their CoP to accommodate for the change in surface with the mean CoP moving 9.4% closer to the front foot in downhill lies and 8.9% closer to the trail foot for uphill lies. Peterson and McNitt-Gray [43] demonstrated how skilled golfers are able to manipulate their stance to accommodate ‘lead leg up’ and ‘lead leg down’ positions, when compared with a more neutral position. Despite these differences, no significant difference in net angular impulse between the three different postures were evident due to golfers typically taking a modified stance to accommodate the shot in either uphill or downhill positions. Kim et al. [37] investigated changes in CoP in the address position in 11 professional golfers, using a 5-iron with varying ball positions (i.e. moving towards and away from the target from the reference or neutral position). When the ball was positioned further back in the stance (i.e. closer to the trail leg), the CoP also shifted towards the trail leg by − 8.4 ± 7.1 mm compared with 11.5 ± 12.2 mm, when the ball was in the reference position. This alteration also changed the vertical GRF of the trail foot 48.0 ± 4.9% of bodyweight compared with 43.2 ± 4.5% of bodyweight in the reference position—indicating that ball position not only changes CoP but also the vertical GRF strategy as well.

Kim and colleagues [5, 38] further investigated the ball position’s impact on GRF in 20 professional golfers. Vertical GRF of the lead foot showed that the left-sided ball positions (positions moved further along the *y*-axis towards the target) were associated with greater magnitudes (full ball width on the left side: + 1.8% of bodyweight; half a ball width on the left side: + 1.0% of bodyweight), whereas the right-sided ball positions (positions moved further away from the target on the *y*-axis) exhibited smaller vertical GRF on the lead foot (full ball width on the right side: − 1.6% of bodyweight; half a ball width on the right side: − 1.1% of bodyweight) when compared with the reference position of 60.7 ± 4.7% bodyweight on the lead foot. Jones et al. [35] used pointwise median absolute deviation (p-MAD) to indicate variability of GRF trajectories. The results indicated no significant difference in GRF strategies between driver and 6-iron swings. However, it should be noted that this study only recruited three golfers, which almost certainly contributed to the lack of statistical findings. In contrast, Peterson et al. [42] demonstrated that the net angular impulse generated by both legs was greater for the driver than for the 6-iron, due to an increase in relative horizontal GRF, in a group of 11 skilled golfers. Somewhat unsurprisingly, these data would indicate that there is greater GRF for driver swings compared with iron swings; however, the individual strategy used by any given golfer may be similar between clubs. This is supported in seminal work by Ball and Best [26, 27], who showed that no single technical model exists for how a golfer interacts with the ground and swings the club.

Jones et al. [36] evaluated 104 amateur golfers using principle component analysis with varied handicaps. They demonstrated that skilled golfers showed reduced variability in their movement strategy (as identified from force platforms) compared with less skilled golfers, when using both a driver and 5-iron. Further to this, McNitt-Gray et al. [40] demonstrated how skilled golfers manipulate GRF to control shot distance. Peak horizontal GRF was reduced in the lead leg by 5% when trying to reduce shot distance to a specific target, compared with a non-modified trial using a 6-iron. McHugh et al. [32] also showed that skilled golfers exhibit an unweighting movement in their lead foot more than unskilled golfers, indicating that a harder push into the floor was evident prior to impact. Queen et al. [44] also showed GRF differences between skilled and less skilled golfers, with skilled golfers demonstrating a greater transfer of vertical GRF from trail to lead leg during the downswing, also producing peak vertical GRF earlier in the downswing than unskilled players. Similarly, Okuda et al. [41] identified GRF differences between skilled and unskilled golfers during different points of the swing. Significant differences between skilled and less skilled players were found for the lead foot vertical GRF during the backswing—skilled: 35 ± 11% bodyweight; less skilled: 25 ± 9% bodyweight, and downswing—skilled: 33 ± 19% bodyweight; less skilled: 59 ± 28% bodyweight, respectively. Vertical GRF for the trail foot was also significantly different between groups during the backswing—skilled: 92 ± 12% bodyweight; less skilled: 76 ± 13% bodyweight. Interestingly, this was in conflict with Worsfold et al. [45], who showed that handicap did not influence changes in vertical GRF when using a driver, 3-iron and 7-iron, between eight skilled players (handicap < 7) and eight less skilled players (handicap 8–14). However, the inability to find any meaningful differences between groups may have simply been a consequence of low sample sizes in each group.

### Directions for Future Research

Given the findings in the present systematic review, there are a number of areas for future research that the authors believe would represent important and logical next steps in furthering our understanding of the biomechanics of the golf swing:

- To date, a number of studies have examined GRF data during the golf swing – some in all three planes of motion. However, the reliability of GRF data is substantially less clear, which would affect how the measures are used or interpreted across repeated swings or sessions. This represents an important step in understanding the usability of GRF data, so that practitioners have an appreciation of what represents a player’s natural bandwidth of variability. In turn, this information would help practitioners comprehend what represents a true change after any kind of coaching or physical training intervention has been employed.
- When reporting GRF data, almost all studies have focused on discrete points during the swing (i.e. peak force). However, while we know that peak force is likely to occur somewhere between club vertical and club horizontal during the downswing [28], it represents a small moment during the swing. Thus, some form of continuous analysis (e.g. statistical parametric mapping) would enable practitioners to better understand ‘periods of relevance’ in the swing and not just discrete points.
- From what the sport science profession already knows about the key drivers behind human locomotion—i.e. impulse [46]—it is surprising to note that virtually no studies have tried to quantify this metric during the golf swing and determine its over-arching relevance to swing speed. Put simply, all biomechanical movement is underpinned by how much force is produced over the time period from which force can be applied [46, 47], and the golf swing is no different. Thus, while force production is critical, it is the time period over which it is expressed which mechanistically explains outcomes in sport. Therefore, as a starting point, it is suggested that the metric of impulse be examined in future golf research, with investigations quantifying its reliability and its discriminative ability between skill levels.
- Given the link that has been identified between CoP and CHS in this review, it seems plausible to suggest that future investigations might consider examining whether changes in CoP strategies are trainable factors for enhancing golfer CHS. Specifically, while alterations to CoP are of course possible (by virtue of changes in technique), it is unclear whether an increased change in CoP velocity would elicit positive adaptations to proxy measures such as CHS and distance off the tee. Thus, this represents a potentially important and interesting line of future research.
- Finally, as mentioned in this review, no single technical model exists for how to swing the golf club. Thus, with some degree of bandwidth for how to swing the club, it seems logical to suggest that some future studies consider both acute and chronic coaching interventions to determine whether significant changes in key outcomes can be impacted, such as distance and accuracy. Naturally, this is what happens in day-to-day practice on the range with golfers who work alongside swing coaches. However, such interventions alongside in-depth biomechanical data would provide a more detailed insight into how changes in the golf swing are achieved.

## Conclusions

Changes in both CoP and GRF represent important factors which contribute to golf performance, as defined by CHS and handicap. The majority of studies have been cross-sectional in design and have examined a range of interesting questions. Of note, force production appears to be greater when swinging a driver compared with irons, and skilled golfers are able to exhibit higher GRF than less skilled golfers, further supporting its importance for being one strategy that golfers can use to generate increased CHS.

## Declarations

### Data Availability

Should readers wish to enquire about the data that have been used to compile this systematic review, please contact Andrew Watson (AW1146@live.mdx.ac.uk) or Dr. Chris Bishop (C.Bishop@mdx.ac.uk).
